# Assessing pulmonary circulation in severe bronchopulmonary dysplasia using functional echocardiography

**DOI:** 10.14814/phy2.14690

**Published:** 2021-01-05

**Authors:** Arvind Sehgal, Douglas Blank, Calum T. Roberts, Samuel Menahem, Stuart B. Hooper

**Affiliations:** ^1^ Monash Newborn Monash Children’s Hospital Monash University Clayton VIC. Australia; ^2^ Department of Paediatrics Monash University Clayton VIC. Australia; ^3^ Paediatric and Fetal Cardiac Units Monash Health Clayton VIC. Australia; ^4^ The Ritchie Centre Hudson Institute of Medical Research Clayton VIC. Australia; ^5^ Department of Obstetrics and Gynaecology Monash University Clayton VIC. Australia

**Keywords:** bronchopulmonary dysplasia, fetal growth restriction, nitric oxide, pulmonary hypertension

## Abstract

Pulmonary hypertension (PH) is common in infants with severe bronchopulmonary dysplasia (BPD) and increases the risk of death. The objectives of this preliminary study were to compare responses of pulmonary circulation parameters to 100% oxygen (O_2_) and inhaled nitric oxide (iNO) in infants with BPD and PH using echocardiography. Responses between fetal growth restriction (FGR) and appropriate for gestational age infants were compared. Ten infants <28 weeks GA at birth were assessed at ≥36 weeks corrected gestation. Baseline echocardiography1 was performed which was repeated (echocardiography2) after 30 minutes of O_2_. After a gap of 2–3 hours, iNO was administered for 15 minutes and echocardiography3 was performed, followed by iNO weaning. The gestation and birthweight of the cohort were 25.9 ± 1.6 weeks and 612 ± 175 g. Assessments were performed at 38.7 ± 1.4 weeks corrected gestational age. Baseline time to peak velocity: right ventricular ejection time (TPV/RVETc) increased from 0.24 ± 0.02 to 0.27 ± 0.02 (O_2_, *p* = .01) and 0.31 ± 0.03 (iNO, *p* < .001), indicating a decrease in pulmonary vascular resistance [PVR]. Baseline tricuspid annular plane systolic excursion (TAPSE) increased from 8.1 ± 0.6 mm to 9.3 ± 0.7 mm (O_2_, *p* = .01) and 10.5 ± 1.1 mm (iNO, *p* = .0004), indicating improved ventricular systolic performance. Percentage change for all parameters was greater with iNO. Significant correlations between cardiac performance and PVR were noted. FGR infants noted higher baseline PVR (TPV/RVETc, 0.21 ± 0.02 vs. 0.25 ± 0.01, *p* = .002), lower ventricular performance (TAPSE, 7 ± 1.2 mm vs. 8.6 ± 6 mm, *p* = .003), and lower percentage change with O_2_ and iNO. A reactive component of pulmonary circulation provides real‐time physiological information, which could rationalize treatment decisions.

## Graphical abstract

Pulmonary vasoreactivity testing with oxygen and inhaled nitric oxide can be performed with echocardiography monitoring. Flow through a patent ductus arteriosus portrays sudden hemodynamic shifts, indicating a reactive pulmonary circulation.

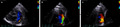

## 1 INTRODUCTION

Bronchopulmonary dysplasia (BPD) remains the most common respiratory sequelae of prematurity. The occurrence of pulmonary hypertension (PH) in this cohort influences survival and significantly increases mortality compared to equally preterm infants with BPD but without PH (An et al., 2010; Bhat et al., 2012; Del Merro et al., 2016; Mourani & Abman, 2013). Hypoxia‐mediated pulmonary vasoconstriction plays an important role in the causation of PH in this cohort (Ajami et al., 2011; Ambalavanan & Mourani, 2014; Bhatt et al., 2001; Coalson, 2006). While this may provide the reversible component (reversed by pulmonary vasodilatation), the remodeling of pulmonary vasculature (due to chronic hypoxia) may contribute to the “fixed” or unresponsive component of PH. Among the prenatal factors, the evolving pattern of BPD also suggests an important pathogenic role for fetal growth restriction (FGR) (Bhatt et al., 2001; Coalson, 2006; Sehgal, Gwini, et al., 2019). The incidence of FGR is high (approximately 27%) among premature births (Sehgal et al., 2019b). Combined, FGR and BPD in the same patient, significantly worsen the clinical and longer‐term respiratory outlook. By way of chronic hypoxia, *in utero* placental insufficiency affects the lung parenchyma *and vasculature* (Maritz et al., 2004, 2005). Data from rats and human infants noted thickened pulmonary arteries indicating the biological plausibility of FGR affecting the pulmonary circulation (Rabinovitch et al., 1979; Sehgal, Gwini, et al., 2019).

While the use of long‐term pulmonary vasodilators in these cohorts is common, the assessment of pulmonary circulation and its reactivity to pulmonary vasodilators before administration may enable risk stratification. Performed with cardiac catheterization (CATH) support, it is a standard approach in many institutions (Abman et al., 2015; Douwes et al., 2016; Sharma et al., 2016). A decline in pulmonary artery (PA) pressure and pulmonary vascular resistance (PVR) indicates a reactive component to PH (Khemani et al., 2007). In a retrospective study, 13/20 (65%) BPD infants demonstrated such response (≥ 20% change in indexed PVR) (Steurer et al., 2019). From the clinical outlook perspective, a responsive circulation is associated with decreased short/long‐term morbidity, contributing to the clinical outlook (Barst, 1986; Douwes et al., 2016; Frank et al., 2019; Sitbon et al., 2005). However, cardiac CATH is an invasive procedure, oftentimes requiring the the transport of critically ill patients. With wide availability of echocardiography, non‐invasive screening for chronic PH is considered a standard of care in many perinatal centers (An et al., 2010; Arjaans et al., 2018; Bhat et al., 2012; Levy et al., 2020; Revanna et al., 2017). Experience in the assessment of right ventricular (RV) performance and PVR opens up possibilities of assessing the pulmonary circulation with echocardiography monitoring.

This study aimed to assess the pulmonary circulation with echocardiography monitoring, comparing hemodynamic responses for 100% oxygen (O_2_) with inhaled nitric oxide (iNO), as well as between FGR and appropriate for gestational age (AGA) infants. Such assessments with echocardiographic guidance in cohorts of BPD‐associated PH have not been reported earlier.

## 2 METHODS

Institutional Human Research Ethics Committee approved this preliminary hypothesis‐generating study (Ref: RES‐19‐0000394L – 53603). After informed written parental consent, 10 infants’ <28 weeks’ GA and severe BPD and PH formed the cohort (severe BPD‐need for positive pressure support and ≥30% O_2_). Normal cardiac anatomy and pulmonary venous connections were documented. Infants were assessed at ≥36 weeks’ corrected GA. FGR was defined as birthweight <10^th^ centile for GA and sex with absent/reversed antenatal Doppler's (Fenton & Kim, 2013). Echocardiography1 was performed with the infant on its baseline respiratory support settings. Echocardiography2 was performed after 30 minutes of 100% O_2_ administered through continuous positive airway pressure (subsequently returned to baseline). After approximately 2–3 hours, iNO was administered through continuous positive airway pressure for 15 minutes at 20 ppm (baseline respiratory support settings). iNO was subsequently weaned over 15 minutes. No pharmacologic sedation was used. The same operator using the Vivid E95 Cardiovascular Ultrasound System (GE Medical Systems, Milwaukee, WI, USA) performed assessments. Previously studied echocardiography parameters were assessed which represented assessments of PVR and RV systolic performance (Czernik et al., 2012; Evans & Archer, 1999; Hayabuchi et al., 2016; Howard et al., 2012; Jain et al., 2014; Koestenberger et al., 2011, 2016; Levy et al., 2015, 2016, 2019; Milnor et al., 1969; Musewe et al., 1990; Patel et al., 2019; Sehgal et al., 2019c, 2020; Ziino et al., 2010). The exact views and cursor position for each assessment and the component of cardiac and pulmonary function assessed has been summarized earlier in our previous publication (Sehgal, Bhatia, et al., 2019). All pulse wave Doppler measurements were calculated from the average of three consecutive cardiac cycles. Left lower pulmonary vein was used uniformly from the “crab‐view.” Time to Peak Velocity/ Right Ventricular Ejection Time (TPV/RVETc) and PA annular peak systolic velocity [s1’] were surrogates to assess PVR. Displacement and velocity of lateral tricuspid annulus in the form of tricuspid annular plane systolic excursion (TAPSE) and tissue Doppler imaging (TDI) systolic velocity [s’] measured RV systolic performance (Badano et al., 2010; Breatnach et al., 2017; Koestenberger et al., 2011). The number of infants having ≥20% change in either of the PVR indices was ascertained.

### 2.1 Statistical analysis

The data are presented as mean ±standard deviation. Baseline data from echocardiography1 were compared with echocardiography2 (100% O_2_) and echocardiography3 (iNO), followed by comparisons between echocardiography2 and echocardiography3 using Student's two‐tailed *t* test. Differences were considered significant if *p* < .05. Pearson product‐moment correlation coefficient, coefficient of determination, the slope of the regression line, and the Y intercept of the regression line were used to describe the relationship between the measures of RV performance and measures of PVR.

## 3 RESULTS

**FIGURE 1:**
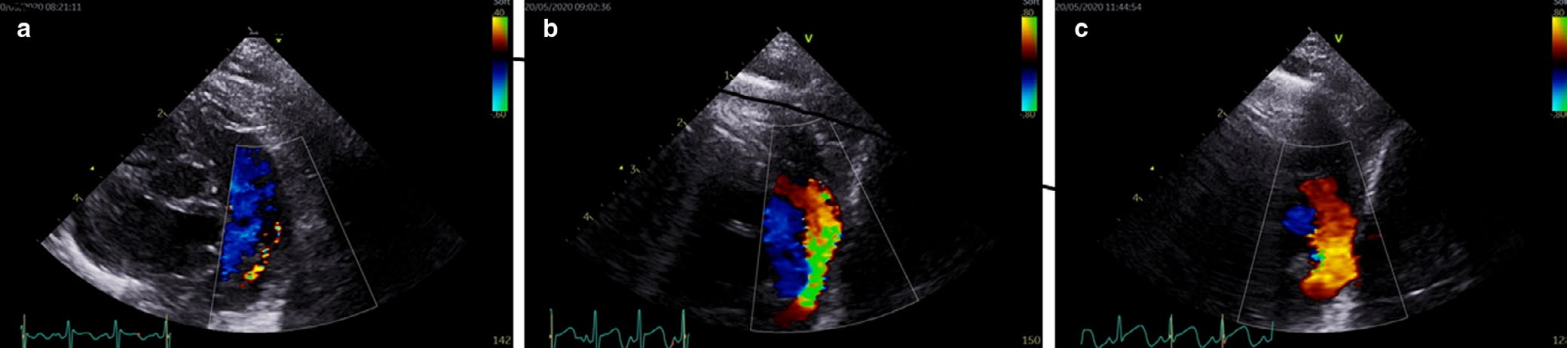
Flow through ductus arteriosus on color Doppler: Baseline (a), 100% oxygen (b), 20 ppm inhaled nitric oxide (C)

**FIGURE 2:**
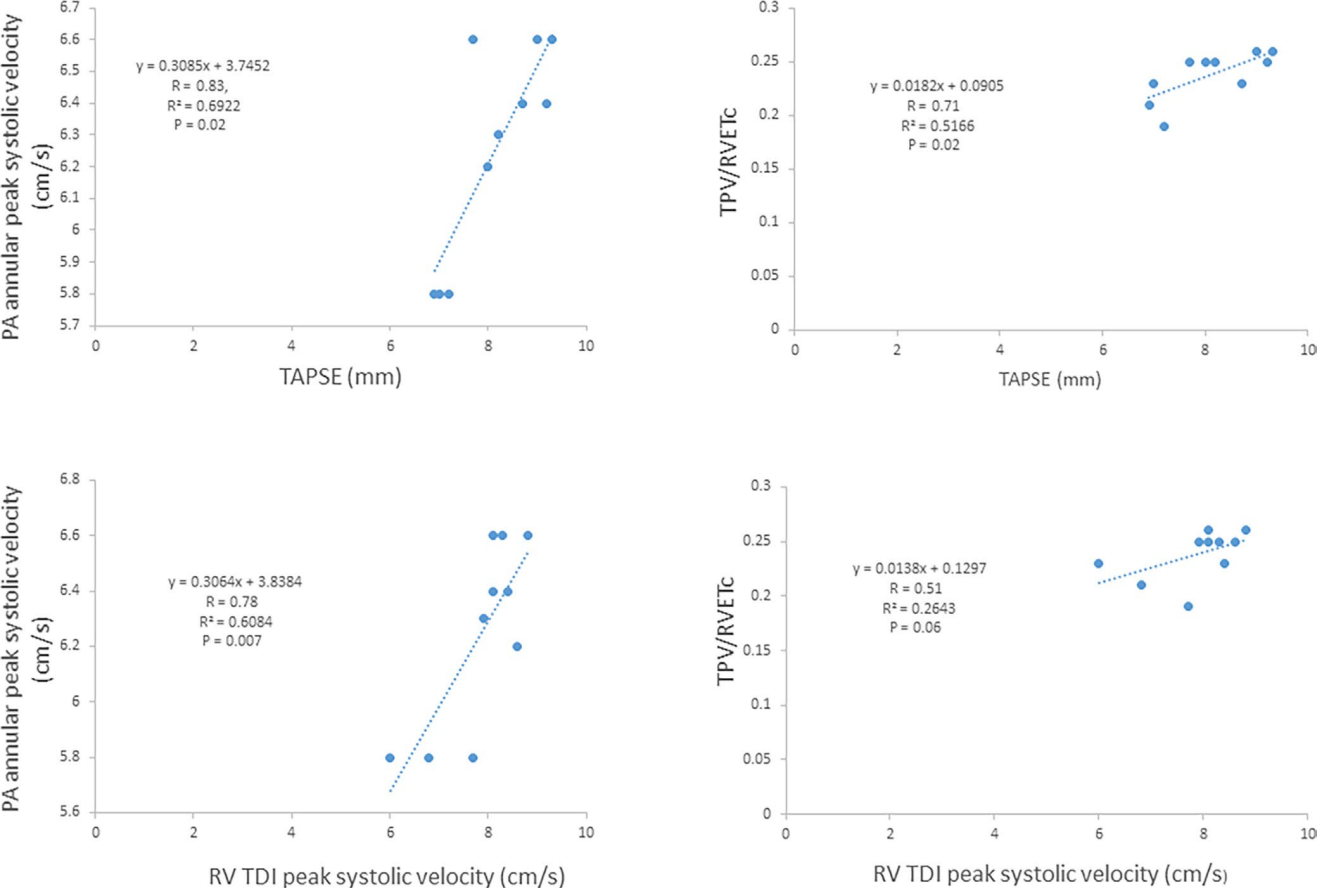
Correlations between RV systolic performance and pulmonary vascular resistance

Table 1 depicts baseline demographic and clinical information. All the infants were receiving non‐invasive ventilation at the time of the study (continuous positive airway pressure). None of the infants were intubated and mechanically ventilated or were on pulmonary vasodilators at the time of the study. Significant improvement in the measures of PVR and RV systolic performance was noted with both O_2_ and iNO, which were accompanied by increased pulmonary blood flow (Table 2). “Percentage change” from baseline was significantly greater with iNO (Table 3). None of the infants dropped PVR by ≥20% with O_2_ (eight infants for iNO) (by TPV/RVETc criteria); the same data for PA annular velocity were one and seven, respectively. Comparing baseline data between FGR and AGA infants, PVR was higher and RV systolic performance significantly lower in the former (Table 4). RV fractional area change (measure of global contractility) was similarly reduced in FGR infants (19.3 ± 0.47% vs. 24.5 ± 1.2%, *p* = .0002). “Percentage change” from baseline was greater in the AGA infants and significantly higher with iNO than O_2_ (Table 5). All (7/7) AGA infants noted a ≥ 20% change to iNO for both parameters, while this was noted 1/3 FGR infants for TPV/RVETc and none for PA annular systolic velocity. One infant (AGA) had a patent ductus arteriosus; increased flow through the duct with O_2_ and iNO is depicted in Figure 1. Significant correlations between the measures of RV performance and PVR as evidence of ventriculo‐arterial coupling were noted (Figure 2). Six infants had measurable tricuspid regurgitation; the maximal velocity at baseline was 2.6 ± 0.4 m/s which changed to 2.4 ± 0.4 with O_2_ (*p* = .5) and to 2.15 ± 0.4 with iNO (*p* = .058), respectively. While not an objective of this study, four infants (all AGA) were later administered sildenafil at the discretion of the medical team. Temporally, this coincided with success in being able to wean respiratory support.

**TABLE 1:** Baseline demographic and clinical parameters (n = 10)

Variable	
Gestational age (weeks)	25.9 ± 1.6
Birthweight (g)	612 ± 175
Apgar score at 5 minutes (median, interquartile range)	8 (7, 9)
Antenatal steroids, n (%)	10 (100)
Mode of delivery, cesarean n (%)	6 (60)
Male sex, n (%)	5 (50)
Fetal growth restriction, n (%)	3 (30)
Postnatal age (days)	92 ± 14
Corrected gestational age (weeks)	38.7 ± 1.4
Weight at study (g)	2350 ± 490
Ventilation mean airway pressure (cm of water)	8.3 ± 1
Oxygen requirement (%)	35 ± 8
Baseline capillary blood gas	
pH	7.3 ± 0.02
pCO_2_	62 ± 5
pO_2_	31 ± 3

* Data presented as mean ± standard deviation, except where indicated otherwise.

**TABLE 2:** Echocardiographic variables after interventions (oxygen [O_2_]/inhaled nitric oxide [iNO]), n = 10

Variable	Baseline	100% O_2_	iNO 20 ppm	*p* (baseline vs. 100% O_2_)	*p* (baseline vs. iNO)	*p* (100% O_2_ vs. iNO)
Pulmonary vascular resistance						
TPV/RVETc	0.24 ± 0.02	0.27 ± 0.02	0.31 ± 0.03	.01	<.001	.02
TDI Pulmonary Artery Annular Peak Systolic Velocity [s1’] (cm/s)	6.2 ± 0.2	7.1 ± 0.5	8.1 ± 0.8	.003	<.001	.2
Right ventricular contractility						
TAPSE (mm)	8.1 ± 0.6	9.3 ± 0.7	10.5 ± 1.1	.01	.0004	.056
TDI tricuspid peak systolic velocity [s’] (cm/s)	7.9 ± 0.6	9.2 ± 0.9	10.3 ± 0.8	.011	<.001	.046
Fractional area change (%)	23 ± 2	27.6 ± 2.7	32 ± 3.5	.005	<.001	.04
*Pulmonary blood flow*						
LPA flow (VTI, cm)	9.9 ± 0.5	11.9 ± 1.1	14 ± 2	.002	.0002	.053
Pulmonary venous flow (VTI, cm)	10.8 ± 0.7	12.2 ± 0.9	13.8 ± 1.5	.01	.0006	.052

Abbreviations: LPA, left pulmonary artery; RVET, right ventricular ejection time; TAPSE, tricuspid annular plane systolic excursion; TDI, tissue Doppler imaging; TPV, time to peak velocity; VTI, velocity time integral.

**TABLE 3:** Comparison of % change from baseline for parameters with 100% oxygen (O_2_) versus inhaled nitric oxide (iNO), n = 10

Variable	100% O_2_	iNO (20 ppm)	*p*
Pulmonary vascular resistance			
TPV/RVETc Reactive pulmonary bed*	13 ± 4 0	23 ± 4 8	<.001
Pulmonary artery annular systolic velocity [s1’] Reactive pulmonary bed*	12 ± 6 1	22 ± 7 7	.006
Right ventricular contractility			
TAPSE	13 ± 2	22 ± 4	<.001
TDI systolic s’ velocity	14 ± 4	24 ± 5	.0001

Abbreviations: TAPSE, tricuspid annular plane systolic excursion; TDI, tissue Doppler imaging, TPV/RVET, time to peak velocity/right‐ventricular ejection time

* Change by ≥20% from the baseline

**TABLE 4:** Comparison of baseline PVR and RV contractility parameters between FGR and AGA cohorts

Variable	FGR (n−3)	AGA (n = 7)	*p* value
Pulmonary vascular resistance			
TPV/RVETc	0.21 ± 0.02	0.25 ± 0.01	.002
Pulmonary artery annular systolic velocity [s1’] (cm/s)	5.7 ± 0.1	6.4 ± 0.15	.0001
Right ventricular contractility			
TAPSE (mm)	7 ± 1.2	8.6 ± 6	.003
TDI systolic s’ velocity (cm/s)	6.8 ± 0.7	8.3 ± 0.3	.002

Abbreviations: AGA, appropriate for gestational age; FGR, fetal growth restriction; TAPSE, tricuspid annular plane systolic excursion; TDI, tissue Doppler imaging; TPV/RVET, time to peak velocity/right‐ventricular ejection time.

**TABLE 5:** Comparison of FGR versus AGA cohorts for % change from baseline with 100% oxygen (O_2_) versus inhaled nitric oxide (iNO)

Variable	FGR 100% O_2_	AGA 100% O_2_	p	FGR iNO	AGA iNO	*p*
Pulmonary vascular resistance						
TPV/RVETc	10.3 ± 2	13.6 ± 4	0.25	19.3 ± 6	25.1 ± 3	.13
Pulmonary artery annular systolic velocity [s1’]	5.3 ± 2	15.1 ± 3.9	0.006	11.6 ± 0.4	25.8 ± 3.9	.0005
Right ventricular contractility						
TAPSE	11.3 ± 2.6	13 ± 1.7	0.32	17 ± 2.1	24.2 ± 2.4	.003
TDI systolic [s’] velocity	9.6 ± 3.8	16.1 ± 1.6	0.01	18 ± 3	24.7 ± 2.7	.015

Abbreviations: AGA, appropriate for gestational age; FGR, fetal growth restriction; TAPSE, tricuspid annular plane systolic excursion; TDI, tissue Doppler imaging; TPV/RVET, time to peak velocity/right‐ventricular ejection time.

## 4 DISCUSSION

Bedside echocardiography facilitated the assessment of pulmonary circulation, and both O_2_ and iNO were significant pulmonary vasodilators, although the percentage change was greater with iNO. FGR status was a clear discriminator, indicating the persistence of *in utero* effects on vascular remodeling.

Assessment of pulmonary circulation in response to O_2_/iNO facilitates risk stratification in BPD (Atz et al., 1999; Krishnan et al., 2017; Mourani et al., 2004). Using CATH, investigators have previously suggested a significant response to be a ≥ 20% drop in mean PA pressure and a decrease in PVR to systemic vascular resistance ratio (Barst, 1986). This has been recently revised to a drop in PA pressure of at least 10 mm Hg (Sitbon et al., 2005). Given this is the first study using echocardiography to compare pulmonary circulation reactivity between iNO versus 100% O_2_, we used the echocardiography PVR indices, while recognizing CATH and echocardiography are two very different modalities. In a study on 26 BPD infants assessed by CATH, a change of ≥20% in PA pressures was noted in nine (35%) infants. This was associated with decreased subsequent mortality risk (Frank et al., 2019). However, unlike in our cohort, approximately half the cohort was already on pulmonary vasodilators. Use of ongoing pulmonary vasodilator therapy ranges from 33–46%, making true interpretation of response difficult (Frank et al., 2019; Steurer et al., 2019). iNO identifies patients who might not be recognized with O2 (Barst et al., 2010; Gan et al., 2014; Hill et al., 2010; Mourani et al., 2004). The dose of iNO used in pediatric cohorts has been variable (10–80 ppm). Both drugs have been generally administered for a variable period (≥10 minutes); in many cases, O2 and iNO have been administered together (Frank et al., 2019; Khemani et al., 2007; Steurer et al., 2019). In spite of limitations, these findings contribute significantly to the clinical outlook and have prognostic significance (Barst, 1986; Douwes et al., 2016; Frank et al., 2019; Sitbon et al., 2005). CATH and bedside echocardiography are performed under very different physiological conditions. The former requires intubation, sedation/analgesia, and anesthesia, which could influence real‐time physiological hemodynamics. It may also reflect the effects of acute changes in lung volume/gas‐exchange during ventilation with anesthesia. Echocardiography assessments are performed in awake infants with no/minimal sedation and may arguably better reflect the physiological state.

### 4.1 Mechanistic linkage

Reduced vessel density, abnormal vascular architecture, and the hypoxic pulmonary vasoconstriction play important roles in PH as well as overall BPD pathophysiology. Reversibility of hypoxic pulmonary vasoconstriction is key but adverse vascular remodeling could blunt the response. Intermittent or chronic hypoxia increase PVR via vasoconstriction, reinforcing the utility of testing the pulmonary vasoconstriction in disease severity assessment, and management of BPD associated PH. The pulmonary arterial vascular smooth muscle cells and alveolar endothelium are targets for O_2_ mediated response. In the main, the vasodilatory effects of iNO are mediated via cyclic GMP, by inhibiting calcium entry into the cell and activation of K+ channels.

### 4.2 Echocardiography facilitates physiological testing of the pulmonary circulation

We utilized previously studied echocardiographic parameters. While both TAPSE and TDI velocities are afterload dependant, all parameters of RV systolic performance (longitudinal [assessed by TAPSE and TDI velocity]) and global (fractional area change]) noted significant changes during pulmonary circulation reactivity testing. This suggests a close inter‐play/ coupling between cardiac contractility and afterload (Figure 2). TPV/RVETc and PA annular peak systolic velocity provide a reliable estimate of invasive PVR and compliance in children (Hayabuchi et al., 2016; Levy et al., 2016); the latter has also been studied in preterm infants receiving surfactant therapy (Sehgal, Bhatia, et al., 2019; Sehgal et al., 2020). Combination of the above indices has been used to characterize ventriculo‐arterial coupling for risk stratification and long‐term monitoring in children with PH (Levy et al., 2016, 2018, 2019). The association between elevated PVR and the RV dysfunction, and its association with sequelae in BPD infants and other clinical situations is known (Blanca et al., 2018; Levy et al., 2019; Schäfer et al., 2018; Sehgal, Gwini, et al., 2019; Sehgal et al., 2016; Yates et al., 2008). A recent study on pediatric PH patients using CATH and echocardiography noted that the Doppler Echo–derived TAPSE/ (TPV: RVET) relationship inversely correlated with invasive systolic pulmonary pressure and PVR (Levy et al., 2018). It is an important prognostic indicator, as pulmonary vascular stiffness predicts mortality in pediatric PH patients (Douwes et al., 2013; Friesen et al., 2019; Schäfer et al., 2018).

### 4.3 Relevant clinical constructs

Sildenafil administration in infants who demonstrated reactivity led to significant clinical improvements (weaning of respiratory support). Identification of such a subset, where reversible pulmonary vasoconstriction is a significant contributor to BPD pathophysiology, has important therapeutic constructs. Pulmonary vasodilators are in variable use for chronic PH although are not approved by the Food and Drug Administration for use in infants. Sildenafil has been used extensively off‐label for the treatment of PH in neonates, infants as well as children. Clinically, such therapies may be administered for months before/after discharge. Pulmonary vasoreactivity testing prior to long‐term oral pulmonary vasodilator treatment may rationalize therapy. Current guidelines do not use such assessments as a determining factor whether a patient should be placed on sildenafil (or other pulmonary vasodilators) or not. Our results note the useful biological plausibility of such a strategy, based on which a physiologic argument of such non‐invasive, bedside testing could be made. Therapeutics commonly used in adult patients should not be simply extrapolated. Pediatric PH, especially in the neonatal population, has many unique clinical features due to complex maturational influences related to lung vascular development and related factors. Physiologic assessments, including cardiac CATH, echocardiography and serum biomarkers, while associated with clinical outcomes, have not been tested as sufficient endpoints for clinical trials. Bedside echocardiography, using well‐studied parameters, opens up the possibilities of precision medicine and physiology driven approach. This is well placed to potentially personalize the care of patients with pre‐term lung disease, ensuring that treatment decisions are based on underlying, demonstrable pathophysiology, and not in a “one‐size fits all approach.” While ductus arteriosus is mostly closed by 36 weeks corrected GA, patency in one infant provided an immediate visual portrayal of sudden hemodynamic shift.

### 4.4 Impact of fetal growth restriction

FGR infants stood out (lower baseline RV systolic performance and elevated PVR) as well as reduced vasoreactivity (percentage change). FGR infants made up a substantial part (46%) of a previous CATH assessed cohort. In another study, FGR infants were significantly more likely to have systemic or supra‐systemic baseline RV pressures than AGA infants were. However, the reactivity of this sub‐cohort was not reported separately in either study (Frank et al., 2019; Khemani et al., 2007). The differential response may be explained by way of muscularization of precapillary vessels, stiff arteries (elastin degradation and its replacement by collagen [100 times stiffer]), reduced endothelial cell function, and impaired eNOS signaling (Gebb & Jones, 2003; McGillick et al., 2016; Rabinovitch et al., 1979). Greater thickening and reduced PA pulsatility are noted in human FGR newborns (Sehgal, Gwini, et al., 2019). Preterm FGR infants also have increased baseline PVR on the first postnatal day (Sehgal et al., 2020). These findings align with the concept of “fetal programming”; disruptions in fetal nutrition/oxygenation may have long‐lasting physiological impact. Lack of reactivity may also indicate that this sub‐cohort may not benefit from long‐term pulmonary vasodilation strategies, but this needs prospective longitudinal analysis.

## 5 CONCLUSIONS

The limitation of small numbers overall and in each group is acknowledged. Infants were not followed up with subsequent echocardiography. Despite limitations, we identified key knowledge gaps that require further study. Assessment of the pulmonary circulation using echocardiography provided real‐time physiological information of the reactivity in response to pulmonary vasodilators. Such assessments may facilitate physiology‐focussed management and pulmonary vasodilator use, providing additional important information for risk stratification and/or response to therapeutic interventions. A prospective study assessing the clinical/echocardiography impact of ongoing vasodilator therapy comparing the responsive/non‐responsive cohorts is better placed to ascertain its role as a therapeutic target. Follow‐up data of survivors of vasodilator therapies are needed to assess effectiveness and long‐term safety.

## CONFLICTS OF INTERESTS

None.
